# Similar polysomnographic pattern in primary insomnia and major depression with objective insomnia: a sign of common pathophysiology?

**DOI:** 10.1186/s12888-017-1438-4

**Published:** 2017-07-28

**Authors:** Matthieu Hein, Jean-Pol Lanquart, Gwénolé Loas, Philippe Hubain, Paul Linkowski

**Affiliations:** 1 Department of Psychiatry and Sleep Laboratory, Erasme Hospital, Université Libre de Bruxelles, ULB, Brussels, Belgium; 2 Department of Psychiatry and Sleep Laboratory, Brussels, Belgium; 3 Route de Lennik, 808-1070, Anderlecht, Belgium

**Keywords:** Major depression, Secondary insomnia, Primary insomnia, Polysomnography, Pathophysiology

## Abstract

**Background:**

Our aim is to verify empirically the existence of a major depressed subgroup with a similar polysomnographic pattern as primary insomnia, including at rapid eye movement sleep level.

**Methods:**

The polysomnographic data from 209 untreated individuals (30 normative, 84 primary insomnia sufferers, and 95 major depressed patients with objective insomnia) who were recruited retrospectively from the Erasme hospital database were studied for the whole night and thirds of the night.

**Results:**

Primary insomnia sufferers and major depressed patients with objective insomnia exhibit a similar polysomnographic pattern both for the whole night (excess of wake after sleep onset, deficit in slow-wave sleep/rapid eye movement sleep, and non-shortened rapid eye movement latency) and thirds of the night (excess of wake after sleep onset at first and last third, deficit in slow-wave sleep in first third, and deficit in rapid eye movement sleep in first and last third), including at rapid eye movement sleep level.

**Conclusion:**

In our study, we demonstrated that major depressed patients with objective insomnia showed a similar polysomnographic pattern as primary insomnia, including at rapid eye movement sleep level, which supports the hypothesis of a common pathophysiology that could be hyperarousal. This opens new avenues for understanding the pathophysiology of major depression with objective insomnia.

## Highlights

- There is a subtype of major depression with deficit in REM and non-shortened REML
- This polysomnographic pattern is similar to that of primary insomnia
- A common pathophysiology related to hyperarousal could explain this similar pattern

## Background

The prevalence of chronic insomnia in the general population ranges from 9.5% to 13.4% [1–3], whereas that of major depression varies from 3.9% to 6.6% with a lifetime prevalence of 9.9% to 16.2% [4–6]. Both insomnia and major depression are important public health problems because they cause absenteeism and significant health care use, along with a very negative impact on quality of life [7–9].

Insomnia and major depression are connected by a particular relationship. Indeed, 90% of depressed individuals have insomnia complaints [10], whereas the insomnia sufferers have a much greater risk of developing depression [11, 12] or committing suicide [13] than non-insomnia sufferers. In polysomnography, primary insomnia and major depression patients have a reduction in sleep efficiency (SE), total sleep time (TST), and slow-wave sleep (SWS), but also an increase in sleep latency (SL), number of awakenings and waking after sleep onset (WASO). However, at the level of rapid eye movement sleep (REM), primary insomnia sufferers (PI) have a deficit and non-shortened REM latency (REML), whereas patients with major depression experience increased REM, shortened REML, and increased REM density [14, 15]. Further, Hubain et al. [16] highlighted in some patients with major depression a sleep reduction affecting both REMS and NREMS in comparable percentages. This difference from the literature can be explained by the fact that in this study, one of the main markers of depression was in fact the absence of sleep, whether observed as long delays prior to entering sleep, or excessive intermittent awakenings that are also characteristics of primary insomnia. Consequently, these patients with major depression presented polysomnographic alterations of objective insomnia type. It is therefore possible that some major depressed patients with objective insomnia (MDOI) show a similar polysomnographic pattern to that of primary insomnia even at REM level.

During sleep, there is a specific distribution of SWS and REM. SWS occurs mainly during the first third of the night and REM in the last third of the night, whereas the second third of the night corresponds to a transition period [17]. In addition, nocturnal biological alterations generally described in primary insomnia and major depression, such as hypothalamic-pituitary-adrenal (HPA) axis, are located mainly at the beginning and end of the night [18–21]. These elements suggest that it would be interesting to study sleep in thirds of the night in these conditions to determine if there are specific polysomnographic alterations in the first and last third of the night where biological alterations are the most important. Moreover, the use of thirds of the night in polysomnographic study is an promising technique, which has been proven useful in the literature [22–26].

The aim of our study is to verify empirically in MDOI the existence of a similar polysomnographic pattern to that of primary insomnia both for the whole night and in thirds of the night. The hypothesis of our study is that there is a major depression subtype with the same polysomnographic alterations (including deficit in REM and non-shortened REML) as primary insomnia, which may indicate the existence of a common pathophysiology. The originality of our study is first to include only MDOI, which will be compared with normative as well as PI groups and second to study of sleep in thirds of the night while considering the natural dynamics of the sleep cycle.

## Materials and methods

### Population

Two hundred nine participants were divided into three groups: a group of 30 normative subjects, a group of 84 PI, and a group of 95 MDOI. These participants were recruited retrospectively from the database of the sleep laboratory of the Erasme Hospital, where 3511 individuals completed sleep laboratory monitoring during the years 2002–2014. The normative subjects present in our database were recruited from prospective studies of our sleep laboratory and only those meeting the inclusion criteria of this study were included in the normative group.

The normative subject inclusion criteria were age ≥ 18 years and lack of the following: sleep pathology, psychiatric disorder, somatic pathology that is severe or may affect sleep, addiction or history of substance abuse, psychotropic or somatic treatment that may affect sleep, and significant daytime sleepiness (Epworth scale score of <10) [27].

The common inclusion criteria for PI and MDOI were age ≥ 18 years and SE < 85% (to include only objective insomnia). The specific inclusion criteria were presence of primary insomnia meeting the diagnostic criteria of the DSM IV-TR [28] for PI, and presence of a major depressive episode meeting the diagnostic criteria of the DSM IV-TR [28] and presence of secondary insomnia meeting the diagnostic criteria of the DSM IV-TR [28] for MDOI.

The common exclusion criteria for PI and MDOI were presence of a sleep disorder other than insomnia or a severe somatic pathology that may affect sleep, use of a psychotropic drug or somatic treatment that may affect sleep, and past or present substance abuse. The specific exclusion criteria were presence of a comorbid psychiatric disorder for PI, and presence of a psychiatric disorder other than major depression for MDOI.

The subjects were free from any iatrogenic cause or somatic pathology that could explain their complaint. Moreover, they were never undergoing treatment; therefore, they did not have to be weaned off medication before polysomnography.

## Methods

### Medical and psychiatric evaluation of participants

Upon admission to the sleep laboratory of the Erasme Hospital, all subjects had their medical records reviewed and a complete somatic check-up performed, including a blood test, electrocardiogram, a daytime electroencephalogram, a urinalysis, and a chest X-ray (only for those over 45 years of age). These steps allowed for a systematic diagnosis of potential somatic pathologies present in people admitted to our unit.

On the day of admission, patients met with a unit psychiatrist who diagnosed subjects with psychiatric disorders using the DSM IV-TR [28] criteria.

On admission, patients completed a series of self-questionnaires for an initial general assessment of their complaints as follows:

- The presence of depressive symptoms was investigated using the Beck Depression Inventory (BDI reduced to 13 items). This scale consists of 13 items that can be scored from 1 to 3. The final score can vary from 0 to 39. A final score of 0–4 indicates an absence of depression, 5–7 a slight depression, 8–15 a moderate depression, and >16 severe depression [29].
- Daytime sleepiness was investigated using the Epworth scale (ESS). This scale consists of eight questions that can be scored from 0 to 3 to assess sleepiness during different daytime situations. The final score varies from 0 to 24. A final score greater than 10 indicates excessive daytime sleepiness [27].
- The presence of insomnia symptoms was investigated using the Insomnia Severity Index (ISI). This index consists of seven questions that can be scored from 0 to 4. The final score can vary from 0 to 28. A score of 0–7 indicates a lack of insomnia, 8–14 subclinical insomnia, 15–21 moderate insomnia, and 22–28 severe insomnia [30].

To avoid missing values, only individuals who responded fully to these questionnaires were included in our study.

### Sleep evaluation and study

A sleep-specific medical record review was carried out systematically by a psychiatrist of the unit on the day of admission. Insomnia was diagnosed after this appointment if the diagnostic criteria for primary insomnia or secondary insomnia, according to the DSM IV-TR [28], were met.

Participants stayed in a sleep laboratory for two nights, including a first night of habituation and a second night of polysomnography from which the data were collected for analysis. For PI and MDOI, polysomnography was performed for evaluation of their complaint of poor sleep. The patients went to bed between 22:00–24:00 and got up between 6:00–8:00, following their usual schedule. During bedtime hours, the subjects were recumbent and the lights were turned off. Daytime naps were not permitted.

Individuals with a sleep pathology other than insomnia, such as hypersomnia, sleep apnea-hypopnea syndrome (index of sleep apnea-hypopnea >5) or periodic limb movement disorder (index of periodic limb movements >5) [31], were excluded from our study. The polysomnographic recordings performed in our unit met the guidelines of the American Academy of Sleep Medicine [32]. The applied polysomnography-montage was as follows: two electro-oculogram channels, three electroencephalogram channels (Fz-Ax, Cz-Ax, and Oz-Ax, where Ax was A1A2 mastoid reference), one submental electromyogram channel, electrocardiogram, thermistors to detect the oro-nasal airflow; finger pulse-oximetry, a microphone to record breathing sounds and snoring, piezoelectric sensors, strain gauges to measure thoracic and abdominal breathing, and leg movement electrodes. Polysomnographic recordings were visually scored by specialized technicians to determine the different stages of sleep as classified by AASM criteria [33] (inter-judge agreement score of 85%).

In order to obtain thirds of the night, as in the study by Hein et al. [23] we divided the sleep period time (defined as the interval separating sleep onset from the last epoch scored as stage 2, SWS, or REM) into three equal parts. SL is thus not included in the first third of the night, and the waking period occurring after the possible early morning awakening is not included in the last third of night. For each third, we calculated the percentages of stage 1, stage 2, SWS, REM, and WASO. The number of ultradian sleep cycles was determined using the following rules: each REMS episode begins with the first epoch of REMS and ends after the last epoch of REMS followed by at least 15 min of NREMS or the final awakening. No minimum duration was required for a REMS episode. A NREMS episode was defined as the time spent between two consecutive REMS episodes, or between a REMS episode and either the beginning or end of the night. An ultradian cycle was defined as the combination of a REMS episode and the immediately preceding NREMS episode, including all awakenings [34].

The polysomnographic data collected were as follows: SL; SPT; TST; SE (defined as TST/time in bed); number of cycles; number of awakenings; duration of each third of the night; the percentages SPT of stage 1, stage 2, stage 3 (SWS), REM, and WASO for the entire night; and the percentages of stage1, stage2, SWS, REM, and WASO for each third of the night.

### Statistical analyses

Statistical analyses were performed using Stata version 14. The normal distribution of the data was verified using histograms, boxplots, and quantile–quantile plots. Since most data followed an asymmetric distribution, we used non-parametric tests for all variables, beginning with the Kruskal-Wallis test to evaluate for significant differences between the medians observed in the normative, PI, and MDOI groups. Subsequently, to adjust our findings to age, gender, and number of cycles (this last only for the NREM and REM analysis by thirds), we used multivariate quantile regression models (based on the median). In these models, polysomnographic data were considered as the dependent variables, whereas age, gender, and number of cycles (this last only for the NREM and REM analysis by thirds) were used as predictors to adjust the regression coefficient for PI vs normative, MDOI vs normative, and PI vs MDOI.

We adjusted our results for age and gender because our samples were not paired for these variables and there are significant changes in sleep depending on age [35, 36] and gender [37, 38] The results for the NREM and REM analysis by thirds of the night were adjusted for the number of cycles because the latter can influence NREM and REM composition in each third. Indeed, the REM/NREM sleep ratio is correlated positively with the number of cycles since REM is more closely associated with the number of cycles than NREM [39].

Results were considered significant when the *p*-value was <0.05 for global tests and <0.05 after Bonferroni correction for multiple comparisons. To facilitate the interpretation and comparison of results with the literature, these results are presented with both their means and standard deviations, in addition to their medians and P25-P75. Correlation analyses were performed using Spearman correlations.

To study the effect of thirds of the night on the percentage of REM, stage 1, stage 2, SWS, or WASO, we have used an analysis of variance for repeated measures with the thirds as the “within” subjects factor and the groups (normative/PI/MDOI) as the “between” subjects factor. An interaction term (between the “within” and the “between” factors) was introduced to view whether the effect of the thirds was significant. To standardize the presentation of our results we present both the means (SD) per third and the corresponding *p*-value (corresponding to the comparison of the means across the thirds) for the whole sample as well as the *p*-values obtained for each group.

## Results

### Demographics

Demographic results are presented in Table 1. The groups were not statistically significantly different in terms of age, gender, or body mass index. PI and MDOI have significantly higher scores in the ISI and the ESS than the normative group. At the BDI, MDOI have significantly higher scores than the normative and PI groups.

**Table 1:** Demographics results

	Mean (SD) Normative (*N* = 30)	Mean (SD) Insomnia (*N* = 84)	Mean (SD) Depression (*N* = 95)	Median (P25-P75) Normative (*N* = 30)	Median (P25-P75) Insomnia (*N* = 84)	Median (P25-P75) Depression (*N* = 95)	*P*-value
Gender (M)	63.33%	58.33%	43.16%				0.053^a^
Age (years)	37.67 ± 11.16	42.38 ± 11.85	43.58 ± 12.37				0.065
BMI (kg/m^2^)	27.24 ± 6.05	25.85 ± 4.88	26.60 ± 6.53				0.480
ISI	4.30 ± 2.60	17.88 ± 2.77	18.82 ± 4.99	5 (3–6)	17 (15–20)	19 (17–23)	<0.001^b^
BDI	1.13 ± 1.48	2.51 ± 1.17	14.59 ± 4.27	0 (0–2)	3 (2–3)	14 (11–17)	<0.001^b^
ESS	5.53 ± 2.39	10.40 ± 5.17	9.94 ± 5.43	6 (4–7)	10 (7–14)	11 (6–13)	<0.001^b^
							^a^Pearson Chi^2^ ^b^Kruskal-Wallis Test

*BMI* Body Mass Index, *ISI* Insomnia Severity Index, *BDI* Beck Depression Inventory, *ESS* Epworth Scale

### Polysomnographic data

#### Sleep onset period and whole night

Polysomnographic results for the sleep onset period and the whole night are shown in Table 2. MDOI and PI had a similar polysomnographic pattern both at the level of the whole night (increased SL and reduced SE, SPT, duration of each third of the night, and TST) and at the level of the sleep architecture (increased WASO, and reduced REM and SWS). There was no significant difference for REM latency. There is a stage 2 deficiency evident only for PI. These differences persist after adjusting results for age and gender (Table 3).

**Table 2:** Polysomnographic results on the entire night

	Mean (SD) Normative (*N* = 30)	Mean (SD) Insomnia (*N* = 84)	Mean (SD) Depression (*N* = 95)	Median (P25-P75) Normative (*N* = 30)	Median (P25-P75) Insomnia (*N* = 84)	Median (P25-P75) Depression (*N* = 95)	*P*-value
SL (min)	17.81 ± 7.82	41.18 ± 37.04	45.57 ± 35.51	18.25 (10.33–24.5)	29.75 (18.42–54.42)	36.50 (23.33–59.00)	<0.001^a,b^
SE (%)	88.63 ± 3.85	71.63 ± 9.64	72.52 ± 11.43	89.11 (86.09–91.87)	73.49 (68.52–77.73)	75.50 (68.57–79.75)	<0.001^a,b^
SPT (min)	480.79 ± 55.48	432.37 ± 60.84	436.67 ± 67.14	482.17 (433.67–517.00)	429.75 (408.50–467.00)	439.33 (402.00–469.33)	0.001^a,b^
Duration of 1^st^, 2^nd^ and 3^rd^ third of the night (min)	160.26 ± 18.49	144.12 ± 20.28	145.56 ± 22.39	160.72 (144.56–172.33)	143.25 (136.17–155.67)	146.44 (134.00–156.44)	0.001^a,b^
TST (min)	447.57 ± 49.66	352.68 ± 62.13	362.59 ± 72.59	457.34 (409.33–479.00)	360.25 (322.42–393.42)	380.33 (335.00–408.33)	<0.001^a;b^
% stage 1	6.80 ± 2.99	6.98 ± 3.65	6.87 ± 3.83	5.82 (4.65–8.62)	7.20 (3.89–9.13)	6.38 (4.34–8.60)	0.923
% stage 2	55.78 ± 6.64	50.76 ± 8.67	51.50 ± 9.14	56.38 (51.47–61.16)	50.93 (45.61–56.00)	53.03 (47.53–57.55)	0.010^a^
% SWS	10.53 ± 6.16	6.92 ± 7.32	7.38 ± 6.85	9.46 (4.08–15.31)	4.64 (0.59–11.34)	5.74 (1.51–11.30)	0.009^a,b^
% REM	20.09 ± 3.21	16.96 ± 5.36	16.59 ± 5.36	19.89 (17.57–22.54)	17.15 (14.11–20.24)	17.14 (12.97–19.42)	<0.001^a,b^
% WASO	6.80 ± 3.37	18.38 ± 10.28	17.65 ± 11.18	6.24 (4.29–8.11)	15.98 (11.94–23.97)	15.99 (10.96–21.54)	<0.001^a,b^
REML	71.49 ± 24.16	85.75 ± 49.82	88.45 ± 57.47	72.5 (53.5–81.5)	69.00 (53.75–109)	70.67 (54.50–101.00)	0.764
Cycles	4.60 ± 0.97	3.83 ± 1.16	3.94 ± 1.20	4.5 (4.00–5.00)	4.00 (3.00–4.00)	4.00 (3.00–5.00)	0.004^a,b^
Number of awakenings	19.53 ± 5.16	36.25 ± 16.04	37.12 ± 21.46	19.00 (17.00–23.00)	29.00 (24.00–45.50)	29.00 (25.00–41.00)	<0.001^a,b^
							Kruskal-Wallis Test

*SL* sleep latency, *SE* sleep efficiency, *SPT* sleep period time, *TST* total sleep time, *SWS* slow-wave sleep, *REM* Rapid eye movement sleep, *WASO* wake after sleep onset, *REML* REM latency

After Bonferroni correction: ^a^Normative vs Insomnia *p* < 0.05, ^b^Normative vs Depression *p* < 0.05, ^c^Insomnia vs Depression *p* < 0.05

**Table 3:** Modifications in polysomnographic results between the groups “Depression”, “Insomnia” and “Normative”, adjusted for age and sex

Normative (*N* = 30) Insomnia (*N* = 84) Depression (*N* = 95)	b_a1_(ES) Group “Insomnia” vs. “Normative”		b_a2_(ES) Group “Depression” vs. “Normative”	b_a3_(ES) Group “Insomnia” vs. “Depression”	*P*-value adjusted for age and sex
SL (min)	16.67 (6.22)^a^		16.69 (6.21)^a^	−0.30 (4.39)	0.018
SE (%)	−15.47 (1.96)^a^		−12.41 (1.96)^a^	3.06 (1.38)	<0.001
SPT (min)	−51.59 (13.25)^a^		−44.35 (13.22)^a^	7.25 (9.35)	0.001
Duration of 1^st^, 2^nd^ and 3^rd^ third of the night (min)	−17.20 (4.42)^a^		−14.78 (4.41)^a^	2.42 (3.12)	0.001
TST (min)	−86.17 (14.32)^a^		−70.33 (14.28)^a^	15.83 (10.10)	<0.001
% stage 1	0.94 (1.04)		0.88 (1.03)	−0.06 (0.73)	0.643
% stage 2	−5.67 (2.16)^a^		−4.40 (2.15)	1.24 (1.52)	0.033
% SWS	−3.94 (1.52)^a^		−3.41 (1.52)^a^	0.52 (1.08)	0.034
% REM	−2.86 (1.21)^a^		−3.36 (1.22)^a^	−0.50 (0.86)	0.021
% WASO	8.14 (2.12)^a^		8.87 (2.11)^a^	0.73 (1.49)	0.001
REML	−0.49 (8.79)		−0.24 (8.77)	0.25 (6.21)	0.998
Cycles	−1.00 (0.18)^a^		−1.00 (0.18)^a^	0.00 (0.13)	<0.001
Number of awakenings	11.31 (3.43)^a^		10.31 (3.43)^a^	−1.00 (2.42)	0.004
				^a^ *p* < 0.05	

b_a1_ (ES)/b_a2_ (ES): quantile regression coefficient adjusted (standard error). These coefficients are the difference of median adjusted for age and gender between “Insomnia”/"Depression” group and the “Normative”

b_a3_ (ES): quantile regression coefficient adjusted (standard error). These coefficients are the difference of median adjusted for age and gender between “Insomnia” group and “Depression” group

*SL* sleep latency, *SE* sleep efficiency, *SPT* sleep period time, *TST* total sleep time, *SWS* slow-wave sleep, *REM* Rapid eye movement sleep, *WASO* wake after sleep onset, *REML* REM latency

#### Thirds of the night

Polysomnographic results for thirds of the night and the analysis of REM, stage1, stage 2, SWS, and WASO are shown in Table 4. At the level of thirds of the night, MDOI and PI had a similar polysomnographic pattern (a REM deficit in the first and last third of the night, a SWS deficit in the first third of the night, a stage 2 deficit in the first and second third of the night, and a WASO excess in the first, second, and last third of the night). These differences persist after adjusting the results for age, gender, and the number of cycles where necessary, except in the second third of the night when excess WASO becomes non-significant in PI and MDOI (Table 5).

**Table 4:** Polysomnographic results for the thirds of the night

	Mean (SD) Whole sample (*N* = 209)	Mean (SD) Normative (*N* = 30)	Mean (SD) Insomnia (*N* = 84)	Mean (SD) Depression (*N* = 95)	Median (P25-P75) Normative (*N* = 30)	Median (P25-P75) Insomnia (*N* = 84)	Median (P25-P75) Depression (*N* = 95)	*P*-value
% REM	(*p* < 0.001)	(*p* < 0.001)	(*p* < 0.001)	(*p* < 0.001)				
1st third	6.97 ± 6.18	10.07 ± 5.23	6.52 ± 6.06	6.29 ± 6.34	10.14 (7.84–14.42)	5.81 (0.00–9.70)	5.76 (0.00–9.73)	<0.001^a,b^
2^nd^ third	17.62 ± 9.57	20.68 ± 10.02	17.37 ± 9.83	16.87 ± 9.11	19.67 (14.16–27.14)	17.80 (10.34–22.91)	17.26 (11.46–22.04)	0.242
3^rd^ third	20.62 ± 10.04	26.57 ± 8.32	20.30 ± 11.02	19.14 ± 9.02	27.17 (22.54–31.59)	19.69 (12.20–27.92)	19.20 (13.69–25.62)	0.001^a,b^
% Stage1	(*p* = 0.003)	(*p* < 0.001)	(*p* = 0.037)	(*p* = 0.172)				
1^st^ third	6.10 ± 3.98	6.02 ± 3.33	6.29 ± 4.40	5.95 ± 3.81	5.07 (3.85–7.41)	5.78 (2.61–9.05)	6.00 (2.90–7.78)	0.971
2^nd^ third	5.76 ± 5.14	5.45 ± 2.92	5.47 ± 4.17	6.11 ± 6.34	4.90(3.10–7.17)	4.52 (2.26–8.55)	5.07 (2.89–7.43)	0.745
3^rd^ third	7.25 ± 7.19	7.91 ± 3.94	6.79 ± 4.43	7.45 ± 9.58	6.79 (4.80–10.09)	5.42 (3.81–8.89)	5.41 (3.68–9.20)	0.169
% Stage2	(*p* < 0.001)	(*p* < 0.001)	(*p* < 0.001)	(*p* < 0.001)				
1^st^ third	39.04 ± 15.96	47.96 ± 10.26	38.99 ± 16.59	36.26 ± 15.97	50.32 (40.52–53.80)	39.33 (29.70–51.08)	38.94 (25.61–47.69)	0.001^a,b^
2^nd^ third	53.13 ± 15.33	61.00 ± 11.03	52.60 ± 13.76	51.12 ± 17.06	62.70 (53.86–70.07)	52.79 (47.07–62.95)	55.27 (42.16–63.73)	0.006^a,b^
3^rd^ third	43.48 ± 13.81	50.11 ± 9.72	40.57 ± 13.38	43.96 ± 14.60	49.87 (43.58–56.77)	42.11 (32.52–50.89)	45.38 (34.56–54.61)	0.003^a^
% SWS	(<0.001)	(<0.001)	(<0.001)	(<0.001)				
1^st^ third	9.96 ± 10.41	20.53 ± 11.34	7.84 ± 8.90	8.50 ± 9.40	19.27 (10.61–29.14)	3.76 (0.26–13.21)	4.67 (0.62–15.36)	<0.001^a,b^
2^nd^ third	7.82 ± 9.97	7.24 ± 7.89	7.94 ± 10.92	7.89 ± 9.77	3.80 (0.93–10.31)	2.03 (0.00–12.52)	3.79 (0.20–12.04)	0.695
3^rd^ third	2.22 ± 4.61	2.37 ± 4.68	1.98 ± 4.60	2.39 ± 4.65	0.52 (0.00–1.92)	0 (0.00–1.75)	0.29 (0.00–2.75)	0.294
% WASO	(*p* < 0.001)	(*p* < 0.001)	(*p* < 0.001)	(*p* < 0.001)				
1^st^ third	37.35 ± 21.33	15.41 ± 7.36	39.11 ± 19.65	42.94 ± 21.48	15.63 (10.09–19.07)	36.59 (27.74–50.01)	38.27 (26.94–55.45)	<0.001^a,b^
2^nd^ third	15.22 ± 18.32	5.63 ± 4.65	15.51 ± 16.80	17.98 ± 21.21	4.68 (2.81–8.10)	12.11 (4.58–19.59)	9.20 (5.42–23.62)	<0.001^a,b^
3^rd^ third	26.10 ± 18.39	12.27 ± 9.43	29.96 ± 18.57	27.05 ± 18.48	8.92 (5.24–15.51)	27.07 (18.18–41.55)	23.78 (13.95–37.67)	<0.001^a,b^
								Kruskal-Wallis Test

*REM* Rapid eye movement sleep, *SWS* slow-wave sleep, *WASO* wake after sleep onset

After Bonferroni correction: ^a^Normative vs Insomnia *p* < 0.05, ^b^Normative vs Depression *p* < 0.05, ^c^Insomnia vs Depression *p* < 0.05

**Table 5:** Modifications in polysomnographic results between the groups “Depression”, “Insomnia” and “Normative”, adjusted for age and gender and the number of cycle when necessary

Normative (*N* = 30) Insomnia (*N* = 84) Depression (*N* = 95)	b_a1_(ES) Group “Insomnia” vs. “Normative”	b_a2_(ES) Group “Depression” vs. “Normative”	b_a3_(ES) Group “Insomnia” vs. “Depression”	*P*-value adjusted for age, sex and the number of cycle
REM (%)				
1^st^ third	−4.71 (1.60)^a^	−4.46 (1.58)^a^	0.25 (1.11)	0.001
2^nd^ third	0.04 (2.86)	−0.71 (2.83)	−0.75 (1.98)	0.923
3^rd^ third	−5.57 (2.57)^a^	−6.27 (2.54)^a^	−0.70 (1.78)	0.046
Stage 1 (%)				
1^st^ third	0.26 (1.08)	0.97 (1.07)	0.72 (0.75)	0.523
2^nd^ third	0.19 (1.18)	0.99 (1.17)	0.80 (0.82)	0.532
3^rd^ third	−1.00 (1.17)	−0.27 (1.15)	0.73 (0.81)	0.566
Stage 2 (%)				
1^st^ third	−8.65 (4.00)^a^	−10.79 (3.96)^a^	−2.14 (2.77)	0.025
2^nd^ third	−10.41 (3.61)^a^	−8.06 (3.58)^a^	2.36 (2.50)	0.017
3^rd^ third	−7.51 (3.65)	−5.13 (3.61)	2.38 (2.53)	0.120
SWS (%)				
1^st^ third	*−11.65* (*2.72*)^a^	−11.48 (2.59)^a^	0.16 (1.88)	<0.001
2^nd^ third	−0.36 (2.26)	0.69 (2.24)	1.05 (1.57)	0.798
3^rd^ third	−0.27 (0.39)	−0.14 (0.38)	0.12 (0.27)	0.766
				*P*-value adjusted for age and sex
WASO (%)				
1^st^ third	22.16 (4.82)^a^	22.10 (4.81)^a^	−0.05 (3.40)	<0.001
2^nd^ third	5.14 (2.76)	2.90 (2.75)	- 2.24 (1.95)	0.157
3^rd^ third	15.69 (4.59)^a^	11.81 (4.58)^a^	- 3.93 (3.24)	0.003
				^a^ *p* < 0.05

b_a1_ (ES)/b_a2_ (ES): quantile regression coefficient adjusted (standard error). These coefficients are the difference of median adjusted for age, gender and the number of cycle when necessary between “Insomnia”/"Depression” group and the “Normative”

b_a3_ (ES): quantile regression coefficient adjusted (standard error). These coefficients are the difference of median adjusted for age, gender and the number of cycle when necessary between the “Insomnia” group and “depression” group

*SWS* slow-wave sleep, *REM* Rapid eye movement sleep, *WASO* wake after sleep onset

#### Effect of the thirds of night on the percentage of different stages of sleep and WASO

The effect of thirds of the night on the percentage of REM, stage 1, stage 2, SWS, or WASO is shown in Table 4. The interaction between the third’s and the group’s factors were significant for SWS (*p* < 0.001) and WASO (*p* = 0.020), but non-significant for stage 1 (*p* = 0.828), stage 2 (*p* = 0.318), and REM (*p* = 0.563). For the whole sample as well in each group, the comparison of the means across the thirds were significant for REM, stage 1, stage 2, SWS, and WASO, except for stage 1 in MDOI.

### Correlations between different stages of sleep and WASO

Correlations between different stages of sleep and WASO are shown in Table 6. For the whole night as well as the first and last third of the night, the percentage of WASO was significantly negatively correlated with the percentage of REM, SWS, and stage 2. There was a higher correlation between WASO and stage 2 than between WASO and REM or SWS. In addition, REML was significantly negatively correlated in PI and MDOI with the percentage of REM in the first third of the night.

**Table 6:** Correlation coefficients between the WASO and REM/SWS/stage 1/stage 2, for the total of the night, 1st/3rd and 3rd/3 thirds of the night

Group insomnia							Group depression						
Total night		1^st^ third			3^rd^ third		Total night		1^st^ third			3^rd^ third	
	% WASO		% WASO	REML		% WASO		% WASO		% WASO	REML		% WASO
% REM	−0.500	% REM	−0.497	−0.397	% REM	−0.607	% REM	−0.577	%REM	−0.631	−0.468	% REM	−0.594
% SWS	−0.396	%SWS	−0.370	/	% SWS	−0.335	% SWS	−0.297	% SWS	−0.447	/	% SWS	−0.377
% stage 2	−0.511	% stage 2	−0.669	/	% stage 2	−0.657	% stage 2	−0.506	% stage 2	−0.726	/	% stage 2	−0.683
% stage1	NS	% stage1	NS	/	% stage 1	NS	% stage 1	NS	% stage 1	−0.310	/	% stage 1	NS

All correlations were significant at *p* < 0.005

*REM* Rapid eye movement sleep, *SWS* slow-wave sleep, *WASO* wake after sleep onset

## Discussion

In our study, we demonstrate that MDOI shows a similar polysomnographic pattern to that of primary insomnia for whole nights and thirds of the night, including at REM level. Our results seem to contradict part of the literature on REM in major depression and indicate the existence of a major depression subtype with potential common pathophysiology with primary insomnia.

In the literature, it is generally accepted that patients with major depression experience increased REM, shortened REML, and increased REM density [40]. However, in our study, we showed a deficit in REM and non-shortened REML in MDOI. This difference can be explained by the fact that in polysomnographic studies of major depression, patients are usually included based on clinical diagnosis rather than on polysomnographic criteria [41, 42]. This recruitment based only on diagnoses of depression scales or diagnostic criteria of international classifications, leads to considerable heterogeneity in samples both in the clinical picture [43] and sleep alterations [31]. This heterogeneity may hide some subtypes of major depression with polysomnographic patterns induced by individual specific pathophysiological mechanisms. We recruited patients with major depression based on DSM IV-TR diagnostic criteria as well as polysomnographic criteria, which resulted in greater uniformity of sleep alterations. This improved uniformity has allowed us to identify a subtype of major depression characterized by a polysomnographic pattern similar to that of primary insomnia. Elements supporting this subtype of major depression exist indirectly in the literature. Indeed, in studies by Hubain et al. [16] and Lopes et al. [44], although the major depressed patients were not recruited based on polysomnographic criteria, they mostly showed more pronounced alterations in sleep, with a resulting polysomnography pattern characterized by REM deficiency. Therefore, MDOI appear to be a specific subtype of major depression whose pathophysiology may be the same as that of primary insomnia.

We further demonstrated an elongated SL, decreased SE, and increased WASO in primary insomnia and MDOI. These polysomnographic alterations can be considered as indirect markers of hyperarousal [45], which is one of the currently proposed theories to explain the physiopathology of primary insomnia and major depression [46–49]. Hyperarousal can be defined as increased emotional, cognitive, and physiological activity that interferes with natural disengagement from the environment and reduces the likelihood of sleep [50]. In major depression, as in primary insomnia, hyperarousal can be divided into three categories [51, 52] that are highly interrelated and occur in the model of chronic insomnia [30]: somatic hyperarousal (characterized by increased activity of autonomic activity and HPA systems) [19, 53–58], cognitive hyperarousal (characterized by greater ruminations while falling asleep) [59, 60], and cortical hyperarousal (characterized by an increase in PET scan nocturnal brain activity and high frequency bands) [61–66]. These elements indicate a state of hypervigilance, and this tendency for hyperarousal is present throughout the 24-h cycle [67]. Thus, hyperarousal provides a reasonable explanation for the tendency to have nighttime awakenings, early morning awakenings, and difficulties with falling asleep, all of which characterize primary and secondary insomnia. However, although there are also elements in favor of hyperarousal in major depression, it would seem that hyperarousal is present only in some patients with major depression, especially those with marked sleep alterations of objective insomnia type. Indeed, some patients with major depression, like those with primary insomnia, show hyperactivity of the HPA axis resulting in failure to suppress cortisol secretion after administration of dexamethasone [68]. In these non-responders, there are polysomnography elements in favor of objective insomnia consistent with hyperarousal: reduced TST, SE, SWS, and increased WASO [69]. Furthermore, alterations in the HPA axis in such individuals are positively correlated with percentages of WASO and stage 1, as they are negatively correlated with percentages of stage 2, SWS, and REM [70]. Since we recruited only MDOI, we showed polysomnographic abnormalities of objective insomnia type consistent with hyperarousal that could be common pathophysiology explaining the existence of a polysomnographic pattern similar to primary insomnia.

In PI and MDOI, we have shown that hyperarousal is associated with an increase in brain activity during NREM and REM, causing a tendency to awaken and greater instability at these sleep stages with consequent an increased WASO, and reduced NREM and REM for the whole night. Indeed, in primary insomnia, at the NREM level, Nofzinger et al. [71] have shown that WASO (subjective and objective) were positively correlated with increased brain activity in a PET scan. In addition, it has been demonstrated that PI showed increased brain activity in the β band during NREM [61], and more precisely during stage 2 [62] and SWS [63]. At the REM level, Merica et al. [72] and Perlis et al. [73] also highlighted more significant β activity during this stage of sleep. On the other hand, patients with major depression have an increased Ω power band at the REM and NREM levels [56]. Furthermore, Nofzinger et al. [74, 75] showed an increase in nocturnal cerebral metabolism during REM and NREM in the PET scan. Moreover, among major depression patients, the activity of the β power band is positively correlated with brain metabolism in the PET scan and negatively to the subjective quality of sleep [76]. Thus, these alterations induced by hyperarousal make it possible to better understand the similar polysomnographic pattern of objective insomnia type between PI and MDOI for the whole night.

At the level of thirds of the night, we have demonstrated that excess WASO occurs during the first and last third of the night, which corresponds to the location of the main biological changes associated with hyperarousal highlighted in PI and MDOI (e.g., in alterations of the HPA axis) [18–21]. The highlighting of this specific dynamics of the polysomnographic alterations related to hyperarousal as well as the particular distribution of the sleep stages during the night (SWS in the first third and REM in the last third of the night) provide a better understanding of the location of deficits in SWS (the first third of the night) and REM (the first and last third of the night). The presence of a similar distribution of polysomnographic alterations during thirds of the night reinforces the theory of hyperarousal as a common pathophysiology in PI and MDOI.

Moreover, in our study, we demonstrated that REML is negatively correlated with the percentage of REM in the first third of the night in PI and MDOI where non-shortened REML can be explained by the presence of a deficiency in REM in the first third of the night. We have also shown that during the first third night, the percentage of REM is negatively correlated with the percentage of WASO, which is an indirect marker of hyperarousal. This non-shortened REML may therefore also be an indirect marker of hyperarousal in view of its potential role in REM deficiency in these patients.

More research is required to confirm our findings concerning the existence of a potential common pathophysiology in MDOI and PI. For example, it might be prudent to study more specific markers of hyperarousal, such as β activity, abnormalities of the HPA axis, or alterations in autonomic activity. Another research approach could relate the polysomnographic alterations highlighted in our study and the daytime consequences of hyperarousal using the multiple sleep latency tests, which is a valid measure of physiological arousal [77].

## Conclusions

In our study, we demonstrated that MDOI had a polysomnographic pattern similar to that of PI for the whole night and thirds of night, which is characterized by a deficit in REM and non-shortened REML. The existence of a common pathophysiology, such as hyperarousal, could explain this similar pattern between primary insomnia and this major depression subgroup.

### Limitations

The results obtained in our study come from retrospective data that, even if they have been encoded in a systematic manner, cannot be verified in most cases directly with the participant, which means that our results need to be replicated in prospective studies. Even if the adjustment of the results by the number of cycles for the NREM and REM analysis by thirds of the night allows for consideration of the effect of the number of cycles on the composition in REM and NREM in each third of the night, one of the limitations of this approach is that it does not make it possible to completely correct the cycles divided between two thirds of the night.

## Abbreviations

AASM: American Academy of Sleep Medicine
APA: American Psychiatric Association
BDI: Beck Depression Inventory
DSM IV-TR: Diagnostic and Statistical Manual of Mental Disorders fourth edition - Text Revision
ESS: Epworth Scale
HPA: Hypothalamic-Pituitary-Adrenal
ISI: Insomnia Severity Index
MDOI: Major Depression with Objective Insomnia
PI: Primary Insomnia Sufferers
REM: Rapid Eye Movement
REML: Rapid Eye Movement Latency
SE: Sleep Efficiency
SL: Sleep Latency
SPT: Sleep Period Time
SWS: Slow-Wave Sleep
TST: Total Sleep Time
WASO: Wake After Sleep Onset
